# Silencing of ASC in Cutaneous Squamous Cell Carcinoma

**DOI:** 10.1371/journal.pone.0164742

**Published:** 2016-10-21

**Authors:** Katharina Meier, Stefan K. Drexler, Franziska C. Eberle, Karine Lefort, Amir S. Yazdi

**Affiliations:** 1 Department of Dermatology, University of Tuebingen, Tuebingen, Germany; 2 Department of Biochemistry, University of Lausanne, Epalinges, Switzerland; 3 Biozentrum, University of Basel, Basel, Switzerland; Sapporo Ika Daigaku, JAPAN

## Abstract

Apoptosis-associated speck-like protein containing a caspase recruitment domain (ASC) is an important adaptor protein for inflammasome activation, mediating the secretion of protumorigenic innate cytokines. However, ASC is also known to trigger apoptosis in tumor cells, acting as a tumor-suppressor gene, which is lost in several human cancers. The aim of this study was to evaluate the clinical significance of ASC in human cutaneous squamous cell carcinoma (SCC). Initially, ASC expression was immunohistochemically evaluated in non-metastic and metastatic SCC. While ASC expression does not correlate with metastatic potential, it correlates with the degree of dedifferentiation. Using methylation specific PCR we were able to demonstrate ASC silencing by promotor specific methylation and impaired inflammasome function in methylated cell lines, linking epigenetic modifications to innate immune activation in keratinocytes. Interestingly, upon ASC restoration by treatment with demethylating agents, we were able to restore AIM2 and NLRP3 activation. In summary, loss of ASC driven tumor development is counterbalanced in the identical cell by the inhibition of pro-tumorigenic inflammation in the tumor cell itself.

## Introduction

Non-melanoma skin cancer is the most common cancer in humans. While basal cell carcinoma (BCC) does not have a precursor and rarely metastasizes, cutaneous squamous cell carcinoma (SCC) is often preceded by an in-situ carcinoma and able to spread to regional lymph nodes and in rare cases to other organs. Histologically, SCCs are graded via the grade of differentiation [1]. The grade of differentiation and the horizontal size of the tumors were considered to be the key determinants of patient outcome [2]. Recent data however determined vertical tumor thickness as the most important prognostic marker for developing metastasis [3].

Besides UV exposure, human papilloma virus (HPV) or chronic inflammation as seen in acne inversa, lichen planus or chronic wounds are known to be the main risk factors for tumor-development [4]. In contrast, immunosuppressed patients have a higher incidence of SCC and a higher risk of metastasis, pointing towards an ambiguous role of inflammation [5].

Important cytokines of early innate immune responses in the skin are TNF and Interleukin (IL)-1 family members [6][7].

The inflammasome adaptor ASC (Apoptosis-associated speck-like protein containing a CARD, PYCARD) is essential for the secretion of IL-1β. IL-1β is cleaved by activated caspase-1. This inflammatory, non-apoptotic caspase is proteolytically processed by cytosolic multiprotein-complexes called inflammasomes. Myeloid cells deficient in ASC show an impaired secretion of IL-1β [8] and for IL-1α [9] after NLRP3- or AIM2-inflammasome activation. However, aside from its role in caspase-1 activation, ASC functions as a tumor suppressor gene. Down-regulation of ASC expression by aberrant methylation was described in numerous cancers, including melanoma and breast cancer.

Recent studies have provided evidence that inflammation can drive tumor development, but the molecular mechanisms converting tissue inflammation into a tumor-growth remain largely elusive. In a mouse-model of inflammation-induced cutaneous SCC, we demonstrated that activated IL-1-signaling promotes cancer development [10]. IL-1 receptor- or caspase-1-deficient mice showed reduced cancer incidence and tumor number compared to wild-type. However, mice deficient for ASC were not protected against cancer development. To differentiate the putative tumor-suppressive role of ASC in the keratinocyte from its tumor-promoting functions as an inflammasome adaptor, mice deficient for the inflammasome adaptor protein ASC exclusively in myeloid cells (ASCf/f-LysM-Cre+) and mice specifically deficient for ASC in keratinocytes (ASCf/fK14-Cre+) were generated. While ASCf/fK14-Cre+ mice developed more tumors than controls, ASCf/f-LysM-Cre+ mice were protected against cancer, suggesting that in contrast to its pro-inflammatory role in myeloid cells, ASC acts as a tumor-suppressor in skin epithelial cells [10].

In this study, we investigate human primary SCCs of various grades of differentiation, tumor-thickness and outcome to determine whether ASC expression correlates with tumor progression or metastasis of the tumor. Further, we aim to determine whether reversing methylation in SCC-tumors might reestablish the capacity of caspase-1 activation. The resulting data might define promotor methylation as an important factor controlling immune activation in cancer cells.

## Materials and Methods

### Patients

72 patients who underwent surgery and were clinically followed up at the Department of Dermatology, University of Tuebingen, Germany, were eligible to be included in the study. Retrospectively, excisions with an identical tumor thickness, but different grades of differentiation in histology were paired. Paraffin embedded formalin fixed tissue of patients with a known history of SCC metastasis were drawn from the files of the Histopathology Unit of the Department of Dermatology, University of Tuebingen, and were matched to an excision from a patient without metastatic disease. As control groups we included biopsies of BCC and condylomata acuminata which were also drawn from the archives of the Histopathology Unit of the Department of Dermatology, University of Tuebingen. This study was approved by our local ethics committee (317/2016BO2). According to the recommendations of our ethics committee, patients whose biopsies were taken between 2012 and 2014 gave written consent. The mean age at diagnosis was 78 years. The mean tumor thickness of the G1 and G3 tumors of SCC was 6.0 mm and 7.7 mm, respectively (Table 1).

**Table 1:** Metastatic and non-metastatic SCC and well (G1) and poorly (G3) differentiated SCC do not differ in age or tumor thickness.

	Age (years)	Invasion (mm)	ASC 0–33%	ASC 34–66%	ASC 67–100%
**All SCC**	78	6,9	21	15	10
**Metastasized**	76	7,4	4	4	5
**Non metastasized**	78	6,8	17	11	5
**G1**	76	6	4	7	7
**G3**	79	7,7	17	8	3

ASC is more frequently expressed in well-differentiated SCC, but ASC stainig does not differ between metastatic and non-metastatic SCC.

### Immunohistochemistry

4μm sections were cut from the paraffin blocks and treated with 10mM sodium citrate buffer for antigen retrieval. ASC was stained with a rabbit anti-human ASC (Adipogene, Switzerland) antibody at a dilution of 1:1000. ASC immunoreactivity was classified into three grades as described previously:Less than 33% positivity of tumor cells (score 1); 34–66% positivity of tumor cells (score 2); 67–100% positivity of tumor cells (score 3)[11].

### Real-time PCR

Total RNA was extracted from primary human keratinocytes which were isolated as described previously [9] or the various SCC cell lines using the RNeasay Kit (Qiagen). After on-column DNA-digest according to the manufacturer´s instruction, RNA concentration was determined using a Nano Drop spectrophotometer (Thermo Scientific). Superscript II Reverse transcriptase (Life Technologies) was used for cDNA synthesis, ASC-expression quantified on a Light Cycler 480 (Roche) instrument with a SYBR-green qPCR master mix (Roche).

#### Bisulfite conversion and Methylation-specific PCR

Genomic DNA was isolated using the DNA Blood and Tissue kit (Qiagen) after proteinase K digestion. DNA yield was measured with a Nano Drop spectrophotometer (Thermo Scientific). Prior to methylation-specific PCR, DNA was bisulfite converted. Utilizing the EZ-DNA Methylation kit (Zymo) DNA was first denatured to then desulphonate DNA for conversion of Cytosine into Uracil. After purification, 2μg of bisulfite-treated DNA was subjected to methylation specific PCR, using EpiMark Taq Polymerase (Zymo) and primers matching targets of either the methylated or unmethylated sequence of ASC [12]. Where indicated, cells were treated with 5-aza-2’-deoxycytidine at a concentration of 2.5 μM or 5 μM for four days. Medium containing 5-aza-2’-deoxycytidine was changed every day. The cells were lysed using a hypertonic lysis buffer and loaded on a gel for Western blot. Secretion of IL-1α and IL-1β from SCC-cell lines was quantified using the Ready-SET-Go ELISA kits (ebioscience) according to the manufacturer´s instructions. Statistical analysis was performed using an unpaired t-test.

## Results

### ASC expression correlates with differentiation, but not with metastasis

**Fig 1:**
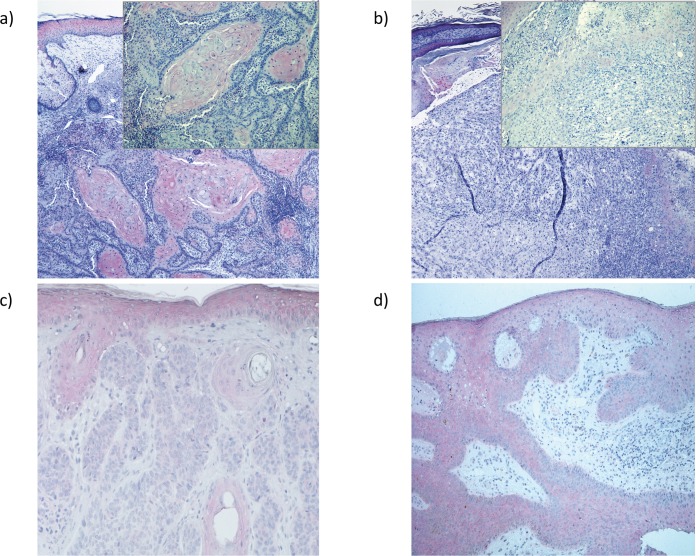
The expression of ASC is reduced de-differentiated (G3) SCC and basal cell carcinoma (BCC), but not in benign HPV-induced condyloma. a) Normal human epidermis and well differentiated SCC express ASC immunohistochemically and as shown in the insert ASC expression is retained in areas of keratinization. while b) ASC expression is lost in de-differentiated G3 tumors, in particular in areas of poor differentiation (insert) and in c) BCC, but not in d) HPV-induced benign condylomata acuminata.

Immunohistochemically, ASC stains the normal epidermis in particular in suprabasal layers, while the basal keratinocytes only show a weak-staining intensity (Fig 1). To evaluate whether ASC-expression might be reduced in cutaneous SCC, SCC of identical tumor thickness, but different grading were stained for ASC. For grading, a modified protocol of Broders using a three tier-grading system was applied. Well differentiated tumors carried <25% (Broders 1) dedifferentiated tumor cells, while poorly differentiated tumors contained > 50% dedifferentiated tumor cells (Broders G3 and G4) [1].

We could observe ASC staining in most of the well-differentiated tumors (Fig 1A). In particular areas with keratinization such as keratin pearls displayed strong immunoreactivity for the staining (Fig 1A). In contrast, areas of dedifferentiation, atypia and lack of proper keratinization express less ASC (Fig 1B).

Interestingly, BCC as the most frequent malignant skin tumor with very rare metastasic potential, lost expression of the tumor-suppressor / inflammasome adapter in all 5 cases analyzed (Fig 1C). In BCC, the tumor cells resemble basal keratinocytes as they arise from the lowest layers of the epidermis or from the outer root sheath of the hair follicle. Keratinization is therefore only rarely present. In contrast, condyloma acuminatum, an HPV-induced benign epidermal hyperplasia arising mainly in the anogenital area did not lose ASC expression in all 5 cases stained (Fig 1D). Condylomata histologically display acanthotic thickening of the epidermis representing benign epithelial tumors in opposition to malignant tumors such as SCC or BCC.

**Fig 2:**
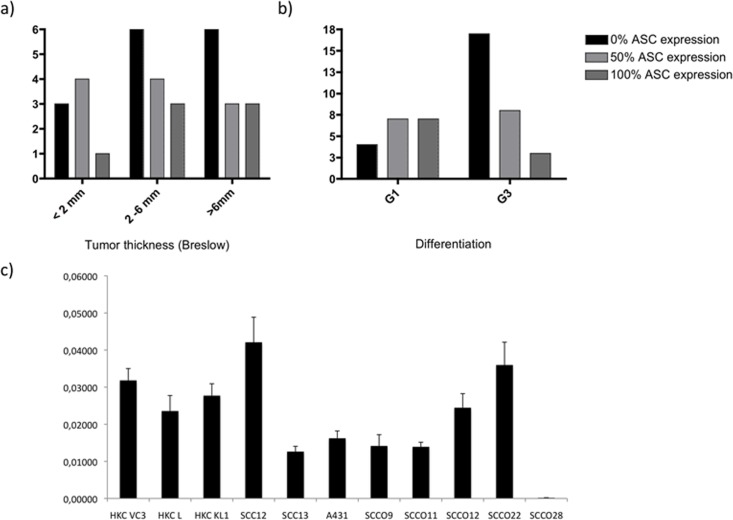
ASC expression does not correlate with tumor thickness, but with the degree of dedifferentiation. Primary cutaneous squamous cell carcinoma (SCC) samples were grouped according to a) tumor thickness and b) grade of differentiation, and ASC staining intensity was evaluated. c) Human primary keratinocytes and various cutaneous or mucosal SCC cell lines were screened for ASC expression by real-time PCR. Primary keratinocytes and 3 cell lines retain ASC expression, while 5 of 8 cell lines display reduced amounts of ASC cDNA.

While increasing vertical tumor thickness is reported to be associated with poorer prognosis [3], ASC immunoreactivity did not correlate with tumor thickness (Fig 2A) or with its metastatic potential (Table 1).

### ASC is silenced in SCC cell lines via promotor-specific methylation

ASC is frequently silenced in malignant epithelial tumors such as BCC and SCC, but is conserved in benign proliferations such as condylomata acuminata. We therefore screened various SCC cancer cell lines and primary human keratinocytes for the expression of ASC. After RNA isolation and cDNA synthesis, the cDNA is subjected to real-time PCR. Compared to primary human keratinocytes, five of eight (62.5%) cutaneous and oral SCC-lines significantly lost ASC expression (Fig 2C).

**Fig 3:**
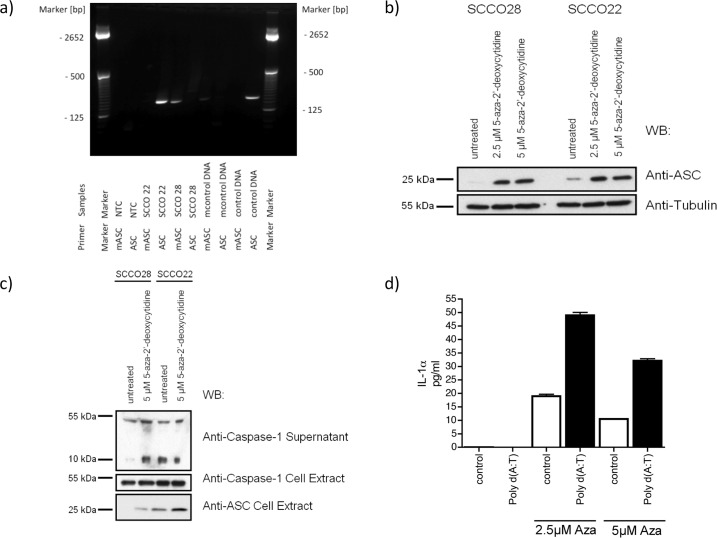
ASC is silenced in squamous cell carcinoma (SCC) cell lines by promotor specific methylation and demethylation restores inflammasome function. a) Bisulfite treated DNA of SCC022 (high expression of ASC) and SCC028 was subjected to methylation specific PCR. In SCC028 only primers detecting the methylated DNA generate a PCR product, indicating that ASC is lost by promotor specific methylation. b) In SCC028 ASC protein expression can be restored by treatment with the demethylating agent 5-aza-2‘-deoxy-cytidine. c) Upon activation with the NLRP3-activator UVB, only SCC022, but not SCC028 cells can cleave caspase-1. SCC028 cells being treated with 5-aza-2‘-deoxy-cytidine restore their capability of inflammasome activation both for c) UVB. Upon treatment with 5-aza-2‘-deoxy-cytidine and d) transfection of dsDNA as activator of the AIM2-inflammasome cells significantly secrete more IL-1α as sign of inflammasome activation (p = 0.0005).

To elucidate the mechanism of how ASC is silenced, a methylation-specific PCR for ASC was established. DNA was isolated from SCC022 which expresses similar amounts of ASC compared to primary keratinocytes, and SCC028 in which ASC is almost entirely absent. Then, DNA was incubated with bisulfite. Bisulfite converts cytosine to uracil, whereas methylated 5-methylcytosine does not react to bisulfite treatment. Therefore, specific primers exclusively amplifying the unmethylated or methylated form of ASC were used. The commercially available fully methylated DNA only amplified using the methylated primer set, while normal DNA only generated a band with the unmethylated ASC-primer pair. A PCR product for ASC in SCC022 (which expresses ASC cDNA) could only be visualized using the unmethylated, but not the methylated primers. As expected, SCC028, which does not express ASC in RT-PCR only amplifies a product with the methylated primer set (Fig 3A). In conclusion, ASC-expression is silenced in SCC via promotor specific methylation.

### Methylated ASC impairs and reversal of methylation restores inflammasome activation in human SCC

5-azacytidine and 5-aza-2’-deoxycytidine are hypomethylating agents which are clinically used in the treatment of myelodysplastic syndrome (MDS). MDS is a precursor of acute myeloid leukemia and is characterized by several aberrantly methylated genes [13]. 5-azacytidine and 5-aza-2’-deoxycytidine are chemical analogues of cytosine. As cytosine is methylated to uracil, azacytidine can substitute for cytosine and can cause hypomethylation and eventually induce de-methylation. ASC expression is diminished by methylation in human SCC and SCC cell lines and methylated ASC might lead to reduced inflammasome activation. It was our aim to determine whether demethylating agents could also restore inflammasome activation. Therefore SCC022 and SCC028 cells were subjected to demethylating 5-aza-2‘-deoxy-cytidine. As expected 5-aza-2‘-deoxy-cytidine restored ASC expression in SCC028 cells (Fig 3B). The cells were then exposed to UVB treatment to activate the NLRP3 inflammasome. Interestingly, SCC028 cells without 5-aza-2‘-deoxy-cytidine treatment could not activate the inflammasome, measured by the secretion of cleaved, activated caspase-1.SCC022 cells, on the other hand, which carry higher endogenous levels of ASC secreted caspase-1 p10 in response to UVB radiation. After demethylation, both SCC028 and SCC022 cells secreted cleaved caspase-1 (Fig 3C). Further, upon AIM2-activation via transfection of poly (dA:dT), IL-1α is only secreted after 5-aza-2‘-deoxy-cytidine exposure (Fig 3D). IL-1α is secreted in an inflammasome dependent manner from both myeloid cells and keratinocytes and can be used as a marker for inflammasome activation in human keratinocytes [14]. Therefore promoter demethylation which is therapeutically used in myeloid leukemia, leads to inflammasome activation, possibly inducing unsuspected pro-inflammatory responses.

## Discussion

As we demonstrated, ASC can, depending on the tissue, have tumor-suppressive or tumor-promotive capacities [10]. In the inflammatory infiltrate ASC impairs IL-1 secretion with IL-1 being a pro-inflammatory, pro-tumorigenic protein, while in the tumor cells ASC serves as a tumor-suppressor. As loss of ASC due to promotor-methylation impairs prognosis in cholangiocarcinoma [15] or lung cancer [16], we aimed to assess whether ASC is differently expressed in metastatic versus non-metastatic primary cutaneous SCC. Comparing SCC of the identical vertical tumor thickness that have or have not metastasized over a follow-up period of at least 4 years, we were not able to detect any difference in staining pattern. In contast to cholangiocarcinoma or lung carcinoma, keratinocytes are known to carry functional inflammasomes [7] [17] which activate caspase-1 to induce IL-1 secretion. Lung epithelial cells have not been reported as inflammasome containing cells. Putatively, IL-1 production of cells carrying ASC in cutaneous SCC might counteract the loss of the tumor-suppressor, leading to balanced proliferation. Additionally, cutaneous SCCs do not metastasize as frequently as lung or gall bladder carcinoma, therefore the ratio of non-metastatic to metastatic SCC is high.

As dedifferentiation is a further prognostic marker in SCC classification, ASC staining was correlated to G1 or G3 cutaneous SCCs. G1 tumors resemble normal squamous epithelia while G3 tumors do not resemble the original keratinocytes. Hallmarks of poor differentiation are a high number of mitotic figures and the lack of keratin pearls as zones of keratinization. Interestingly, ASC was retained in G1 tumors being able to form keratin, while it was lost more frequently in dedifferentiated G3 tumors. These dedifferentiated tumors often resemble mesenchymal tumors or scar tissue and no longer carry ASC. As ASC alters proliferation by regulation of p53 activation [10] and p53 regulates epithelial-mesenchymal transition (EMT) [18], ASC might regulate the EMT-like dedifferentiation of keratinocytes. Condylomata acuminata, benign HPV-induced proliferations, showed the identical expression of ASC as the epidermis, while BCCs lost the tumor suppressor. BCCs very often silence p53-dependent genes by methylation [19], although they hardly ever metastasize. They originally never show any signs of keratinization as their cells of origin are located near in basal keratinocytes or the hair follicle. After having demonstrated that ASC is silenced in SCC by promotor specific methylation, we screened SCC cell lines for the expression of ASC. Here, some cell lines lost ASC, while others show the identical levels compared to primary keratinocytes. This reflects the clinical presentation, as some SCCs grow slowly, while others grow rapidly. In comparing a cell line that has lost ASC to a cell line where ASC expression is not altered, we were able to restore ASC expression by treatment with a demethylating agent, which is used in the treatment of myeloid leukemia. As expected, ASC was demethylated and became detectable on cDNA and protein levels. Interestingly, functional ASC was also restored after treatment. The tumor cells that silenced ASC were not able to secrete IL-1 or cleave caspase-1. However, after ASC restoration by 5-aza-2‘-deoxy-cytidine the cells responded upon inflammasome activation with caspase-1 cleavage and IL-1 secretion. Therefore, we could for the first time demonstrate that promotor methylation influences inflammasome activation. This could be of therapeutic relevance, as some treatment options for early epithelial skin cancer demand immune activation. Imiquimod, which is used for superficial SCC and BCC and genital warts, is a ligand for both TLR7 and NLRP3, therefore inducing both IL-1 and interferon. Futhermore, the anti-cancer effect of ingenol mebutate which is used for the treatment of cutaneous SCC in situ is mediated via IL-1 [20].

In conclusion, ASC expression cannot be exploited as a prognostic marker for metastasis, but serves as an indicator for highly differentiated tumors. The dual capacity of ASC being an inflammasome adaptor regulating caspase-1 and IL-1 activation, but also controlling proliferation is restored in cutaneous SCC. The tumor cells can only activate pro-tumorigenic IL-1 if the tumor suppressor ASC is expressed. Therefore, further studies are needed to enhance the understanding of inflammasome regulation in tumor cells via methylation and de-methylation of target genes.
